# Identification of Hub Genes Associated With Development of Head and Neck Squamous Cell Carcinoma by Integrated Bioinformatics Analysis

**DOI:** 10.3389/fonc.2020.00681

**Published:** 2020-05-22

**Authors:** Chia Ying Li, Jia-Hua Cai, Jeffrey J. P. Tsai, Charles C. N. Wang

**Affiliations:** ^1^Department of Surgery, Show Chwan Memorial Hospital, Changhua, Taiwan; ^2^Ph.D. Program in Tissue Engineering and Regenerative Medicine, National Chung Hsing University, Taichung, Taiwan; ^3^Department of Bioinformatics and Medical Engineering, Asia University, Taichung, Taiwan

**Keywords:** head and neck squamous cell carcinoma, differential gene expression analysis, weighted gene co-expression network analysis, the differential co-expression genes, biomarkers

## Abstract

Improved insight into the molecular mechanisms of head and neck squamous cell carcinoma (HNSCC) is required to predict prognosis and develop a new therapeutic strategy for targeted genes. The aim of this study is to identify significant genes associated with HNSCC and to further analyze its prognostic significance. In our study, the cancer genome atlas (TCGA) HNSCC database and the gene expression profiles of GSE6631 from the Gene Expression Omnibus (GEO) were used to explore the differential co-expression genes in HNSCC compared with normal tissues. A total of 29 differential co-expression genes were screened out by Weighted Gene Co-expression Network Analysis (WGCNA) and differential gene expression analysis methods. As suggested in functional annotation analysis using the R clusterProfiler package, these genes were mainly enriched in epidermis development and differentiation (biological process), apical plasma membrane and cell-cell junction (cellular component), and enzyme inhibitor activity (molecular function). Furthermore, in a protein-protein interaction (PPI) network containing 21 nodes and 25 edges, the ten hub genes (S100A8, S100A9, IL1RN, CSTA, ANXA1, KRT4, TGM3, SCEL, PPL, and PSCA) were identified using the CytoHubba plugin of Cytoscape. The expression of the ten hub genes were all downregulated in HNSCC tissues compared with normal tissues. Based on survival analysis, the lower expression of CSTA was associated with worse overall survival (OS) in patients with HNSCC. Finally, the protein level of CSTA, which was validated by the Human Protein Atlas (HPA) database, was down-regulated consistently with mRNA levels in head and neck cancer samples. In summary, our study demonstrated that a survival-related gene is highly correlated with head and neck cancer development. Thus, CSTA may play important roles in the progression of head and neck cancer and serve as a potential biomarker for future diagnosis and treatment.

## Introduction

Head and neck squamous cell carcinoma (HNSCC) is one of most common types of cancer in the world. HNSCC includes several malignancies that originate in the mouth, nasopharynx, oropharynx, hypopharynx, larynx, and neck (1). According to the published global cancer statistics report, there were more than an estimated 650,000 new cases and 330,000 deaths diagnosed in 2018 (2). Many lifestyle factors have been investigated, with tobacco use, alcohol consumption, human papillomavirus (HPV), and Epstein-Barr virus (EBV) infection being considered as the risk factors that are associated with the progression of HNSCC (3). However, HPV is currently the one most well-studied and frequently used biomarker in HNSCC (4–6). In the past several years, the treatments for managing head and neck cancer included the following: radiation therapy, surgery, and chemotherapy. Appropriate combinations of the three treatment modalities is selected according to the site of the cancer and the stage of the disease (1, 3). Although there are diverse treatments for HNSCC, patients have a limited survival advantage.

With the development of genomic technologies, bioinformatics has become increasingly popular for gene expression profiles analysis to study the molecular mechanisms of diseases and discover disease-specific biomarkers (7). One important method to understand the gene function and gene association from genome-wide expression is Weighted Gene Co-expression Network Analysis (WGCNA) (8). WGCNA can be used to detect co-expression modules of highly correlated genes and interested modules associated with clinical traits (9), providing great insight into predicting the functions of co-expression genes and finding genes that play key roles in human diseases (10–12). Furthermore, another powerful analysis within transcriptomics is differential gene expression analysis, which provides methods for studying molecular mechanisms underlying genome regulation and discovering quantitative changes in expression levels between experimental groups and control groups (13). Such gene expression differences can lead to the discovery of potential biomarkers for a particular disease. Therefore, using two approaches, the findings from WGCNA and differential gene expression analysis are combined to enhance the discriminating ability of highly related genes that are useful to serve as candidate biomarkers.

In this study, the mRNA expression data of HNSCC from the TCGA and GEO databases were analyzed by WGCNA and differential gene expression analysis to obtain differential co-expression genes. We further explored HNSCC development through functional enrichment and protein-protein interaction (PPI) analysis combined with survival analysis. The study provides a potential basis to understand the cause and potential molecular events of HNSCC by analyzing differential co-expression genes for clinical diagnosis or treatment.

## Materials and Methods

**Figure 1:**
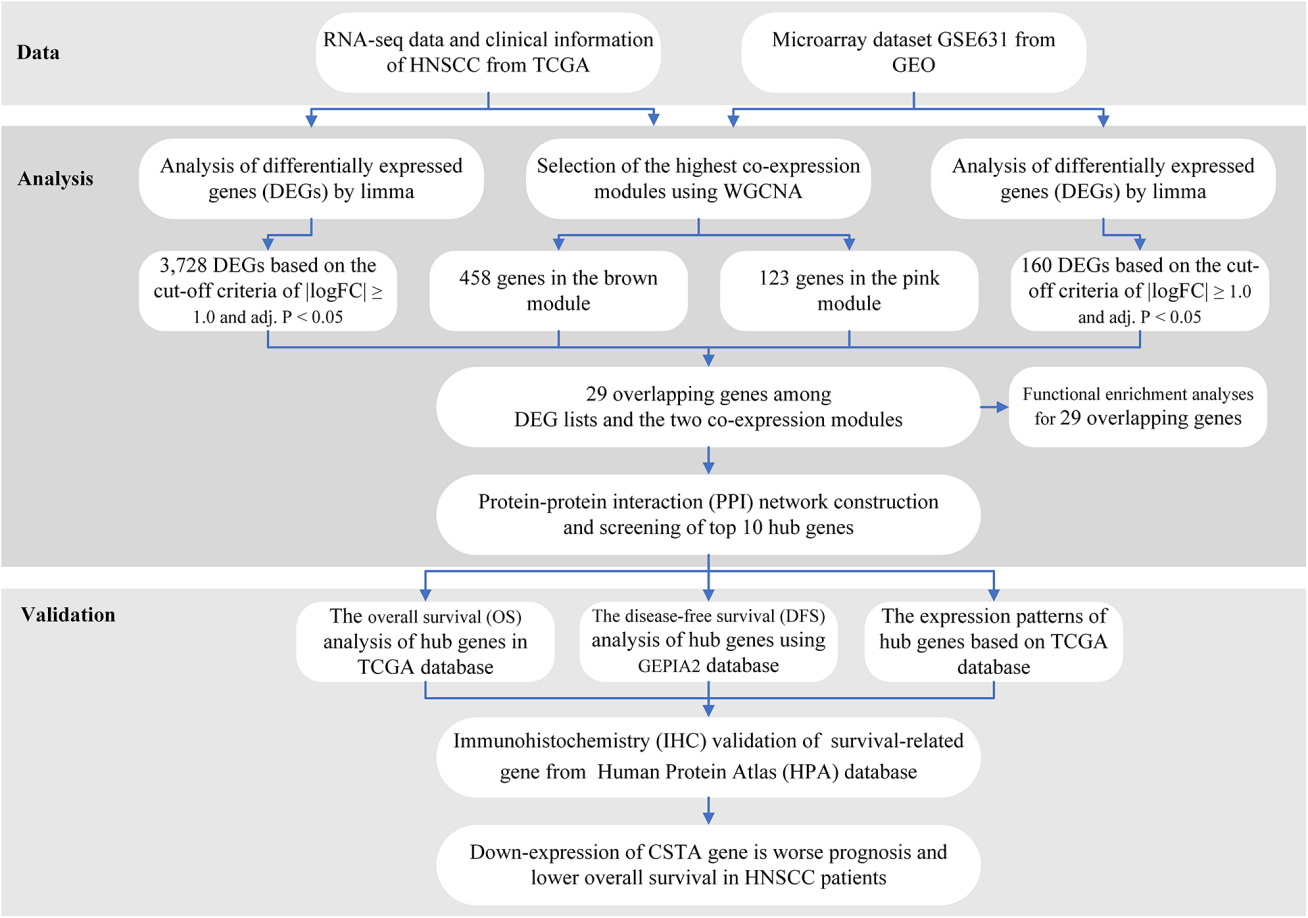
Study design and workflow of this study.

The workflow of the analysis hub gene extraction curation pipeline is shown in Figure 1.

We elaborate on each step in the following sub-sections.

### Datasets From TCGA and GEO Database

The gene expression profiles of HNSCC were downloaded from TCGA (https://portal.gdc.cancer.gov/) and GEO (https://www.ncbi.nlm.nih.gov/gds). In the TCGA database, all data on HNSCC and corresponding clinical information were freely downloaded by R package *TCGAbiolinks* (14). There were 544 NHSCC samples, including 500 head and neck cancers and 44 normal tissues, and RNAseq count data on 19,430 genes. A total of the data had been generated by using the Illumina HiSeq 2,000 platform, and were annotated to a reference transcript set of Human hg38 gene standard track. As suggested by the *edgeR* package tutorial (15), genes of low read counts are usually not of interest for further analysis. So, we kept the genes with a cpm (count per million) ≥1 in this study. After filtering using function *rpkm* in *edgeR* package, which is calculated by dividing gene counts by gene length, a total of 15,367 genes with RPKM values were subject to our next analysis.

In addition, the normalized expression profiles of GSE6631, another gene expression profile of HNSCC from GEO, was obtained using R package *GEOquery* (16). GSE6631 consisted of 22 tumor samples and 22 paired normal tissues from patients with HNSCC, which were studied with the GPL8300 platform [HG_U95Av2] Affymetrix Human Genome U95 Version 2 Array. Probes were converted to the gene symbols based on a manufacturer-provided annotation file and duplicated probes for the same gene were removed by determining the median expression value of all its corresponding probes. As a result, a list of 9,203 genes were selected for the subsequent analysis.

### Identification of Key Co-expression Modules Using WGCNA

Co-expression networks facilitate methods on network-based gene screening that can be used to identify candidate biomarkers and therapeutic targets. In our study, the gene expression data profiles of TCGA-HNSCC and GSE6631 were constructed to gene co-expression networks using the *WGCNA* package in R (8). *WGCNA* was used to explore the modules of highly correlated genes among samples for relating modules to external sample traits. To build a scale-free network, soft powers β = 3 and 20 were selected using the function *pickSoftThreshold*. Next, the adjacency matrix was created by the following formula: a_ij_ = |S_ij_|^β^ (a_ij_: adjacency matrix between gene i and gene j, S_ij_: similarity matrix which is done by Pearson correlation of all gene pairs, β: softpower value), and was transformed into a topological overlap matrix (TOM) as well as the corresponding dissimilarity (1-TOM). Afterwards, a hierarchical clustering dendrogram of the 1-TOM matrix was constructed to classify the similar gene expressions into different gene co-expression modules. To further identify functional modules in a co-expression network, the module-trait associations between modules, and clinical trait information were calculated according to the previous study (17). Therefore, modules with high correlation coefficient were considered candidates relevant to clinical traits, and were selected for subsequent analysis. A more detailed description of the WGCNA method was reported in our previous study (17).

### Differential Expression Analysis and Interaction With the Modules of Interest

The R package *limma* (linear models for microarray data) provides an integrated solution for differential expression analyses on RNA-Sequencing and microarray data (18). In order to find the differentially expressed genes (DEGs) between HNSCC and normal tissues, *limma* was applied in the TCGA-HNSCC and GSE6631 dataset, respectively, to screen out DEGs. The *p*-value was adjusted by the Benjamini–Hochberg method to control for the false discovery Rate (FDR). Genes with the cut-off criteria of |logFC| ≥ 1.0 and adj. *P* < 0.05 were regarded as DEGs. The DEGs of the TCGA-HNSCC and GSE6631 dataset were visualized as a volcano plot by using the R package *ggplot2* (19). Subsequently, the overlapping genes between DEGs and co-expression genes that were extracted from the co-expression network were used to identify potential prognostic genes, which were presented as a Venn diagram using the R package *VennDiagram* (20).

### Functional Annotation for Genes of Interest

To explore Gene Ontology (GO) of selected genes, R package clusterProfiler package (21) was used to explore the functions among genes of interest, with a cut-off criterion of adjusted *p* < 0.05. GO annotation that contains the three sub-ontologies—biological process (BP), cellular component (CC), and molecular function (MF)—can identify the biological properties of genes and gene sets for all organisms (22).

### Construction of PPI and Screening of Hub Genes

In our study, we used the STRING (Search Tool for the Retrieval of Interacting Genes) online tool, which is designed for predicting protein–protein interactions (PPI), to construct a PPI network of selected genes (23). Using the STRING database, genes with a score ≥ 0.4 were chosen to build a network model visualized by Cytoscape (v3.7.2) (24). In a co-expression network, Maximal Clique Centrality (MCC) algorithm was reported to be the most effective method of finding hub nodes (25). The MCC of each node was calculated by CytoHubba, a plugin in Cytoscape (25). In this study, the genes with the top 10 MCC values were considered as hub genes.

### Verification of the Expression Patterns and the Prognostic Values of Hub Genes

In order to confirm the reliability of the hub genes, we verified the expression patterns of the hub genes in different pathological tumors and normal tissues. The expression level of each hub gene between cancer and normal tissue was plotted as a box plot graph. Based on the data from the TCGA database, Kaplan–Meier univariate survival analysis was performed by using the *survival* package in R software to explore the relationship between overall survival (OS) and hub genes in patients. Moreover, the association between disease-free survival (DFS) and hub genes expressed in HNSCC patients was determined using the online tool GEPIA2 (26). In our study, only patients with completed follow-up times were selected for survival analysis and then divided into two separate groups based on the median expression value of hub genes. The survival-related hub genes with log-rank *p* < 0.05 were regarded as statistically significant.

### Validation of Protein Expressions of Survival-Related Hub Genes by the HPA Database

The protein expression of the survival-related genes between HNSCC and normal tissues was determined using immunohistochemistry (IHC) from the Human Protein Atlas database (HPA, https://www.proteinatlas.org/). HPA is a valuable database that provides a large amount of transcriptomics and proteomics data in specific human tissues and cells for researchers (27). Moreover, the IHC-based protein expression pattern is the most common application of immunostaining to detect the relative location and abundance of proteins (28).

## Results

### Construction of Weighted Gene Co-expression Modules

**Figure 2:**
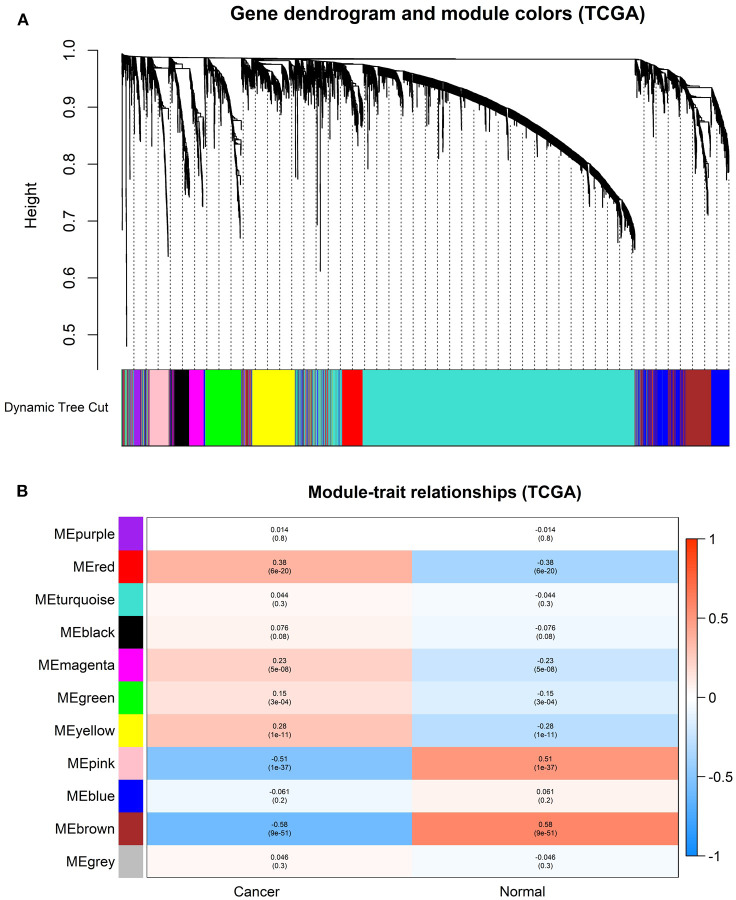
Identification of modules associated with the clinical information in the TCGA-HNSCC dataset. **(A)** The Cluster dendrogram of co-expression network modules was ordered by a hierarchical clustering of genes based on the 1-TOM matrix. Each module was assigned different colors. **(B)** Module-trait relationships. Each row corresponds to a color module and column corresponds to a clinical trait (cancer and normal). Each cell contains the corresponding correlation and *P*-value.

**Figure 3:**
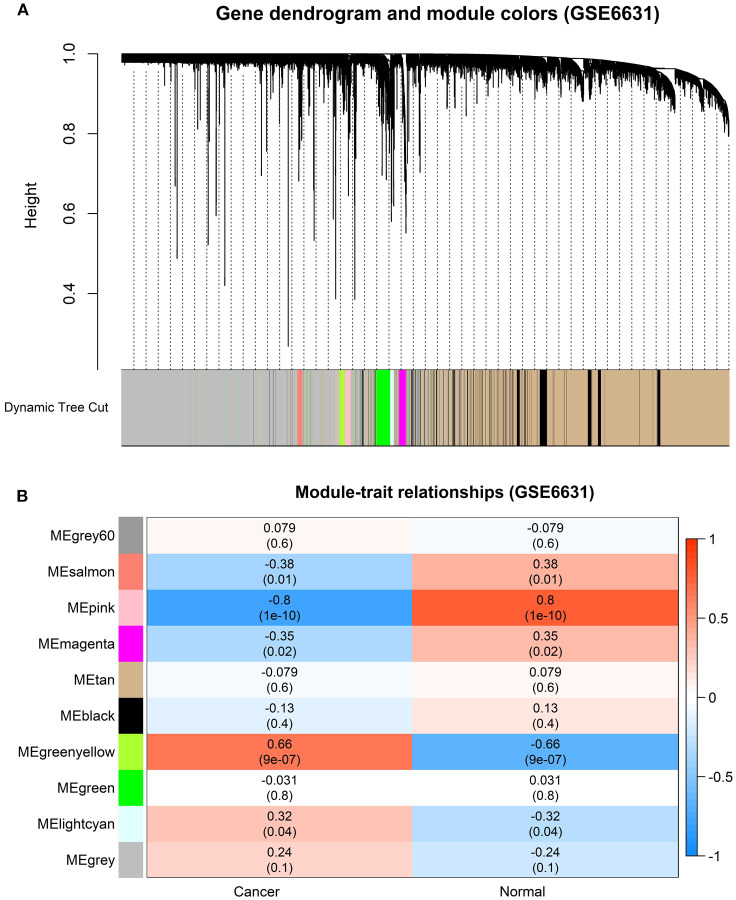
Identification of modules associated with clinical information in the GSE6631 dataset. **(A)** The Cluster dendrogram of co-expression network modules was ordered by a hierarchical clustering of genes based on the 1-TOM matrix. Each module was assigned different colors. **(B)** Module-trait relationships. Each row corresponds to a color module and each column correlates to a clinical trait (cancer and normal). Each cell contains the corresponding correlation and *P*-value.

In order to find the functional clusters in HNSCC patients, the gene co-expression networks were constructed from the TCGA-HNSCC and GSE6631 datasets with the *WGCNA* package. With each module assigned a color, a total of 10 modules in the TCGA-HNSCC (Figure 2A) and nine modules in the GSE6631 (Figure 3A) were identified in the present study (excluding a gray module that was not assigned into any cluster). Then, we plotted the heatmap of module-trait relationships to evaluate the association between each module and two clinical traits (cancer and normal). The results of the module-trait relationships are presented in Figure 2B, 3B, revealing that the brown module in the TCGA-HNSCC and pink module in the GSE6631 were found to have the highest association with normal tissues (brown module: *r* = 0.58, *p* = 9e−51; pink module: *r* = 0.8, *p* = 1e−10).

### Identification of Genes Between the DEG Lists and Co-expression Modules

**Figure 4:**
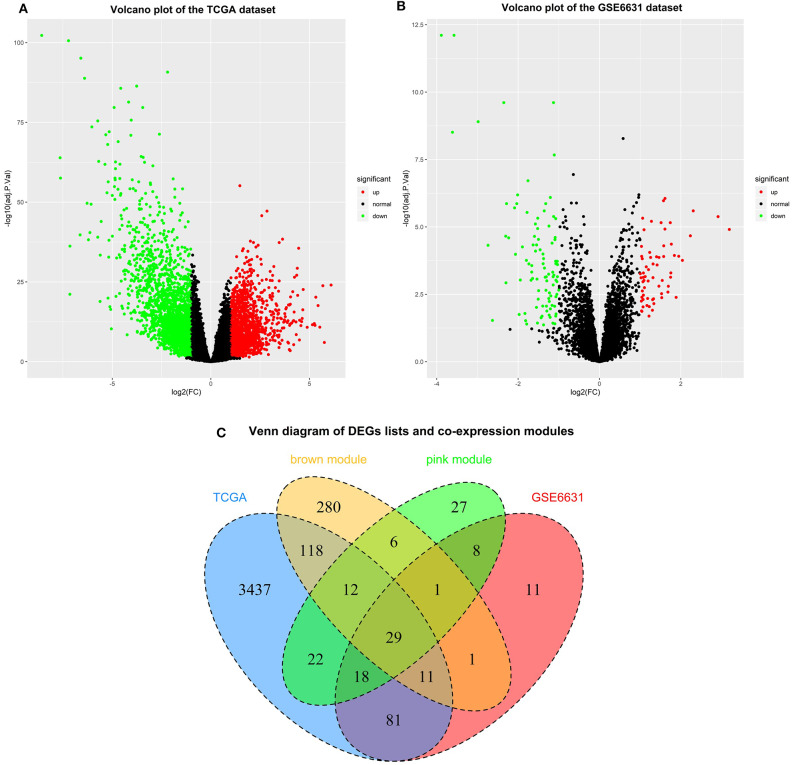
Identification of differentially expressed genes (DEGs) among the TCGA and GSE6631 datasets of HNSCC with the cut-off criteria of |logFC| ≥ 1.0 and adj. *P* < 0.05. **(A)** Volcano plot of DEGs in the TCGA dataset. **(B)** Volcano plot of DEGs in the GSE6631 dataset. **(C)** The Venn diagram of genes among DEG lists and co-expression module. In total, 29 overlapping genes in the intersection of DEG lists and two co-expression modules.

Based on the cut-off criteria of |logFC| ≥ 1.0 and adj. *P* < 0.05, a total of 3,728 DEGs in the TCGA dataset (Figure 4A) and 160 DEGs in the GSE6631 dataset (Figure 4B) were found to be dysregulated in tumor tissues by the *limma* package. As shown in Figure 4C, 458 and 123 co-expression genes were found in the brown module of TCGA dataset and the pink module in GSE6631, respectively. In total, the 29 overlapping genes were extracted for validating the genes of co-expression modules (Figure 4C).

### Functional Enrichment Analyses for the 29 Genes

**Figure 5:**
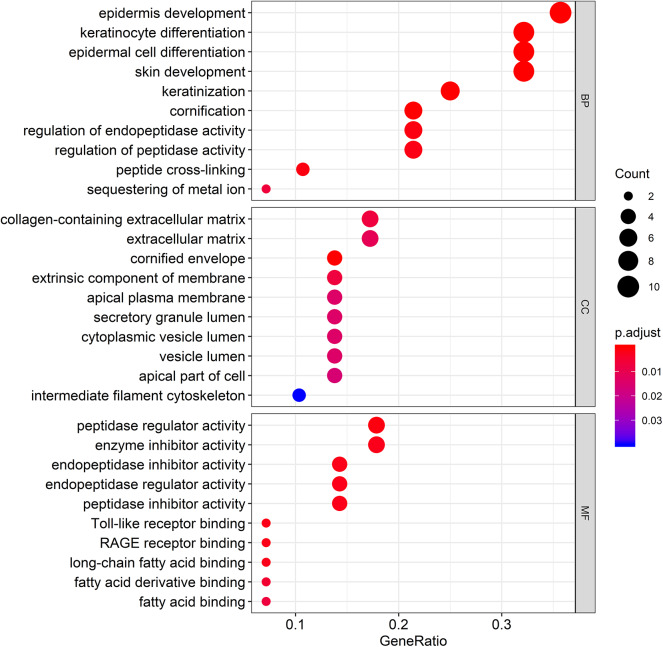
Gene Ontology (GO) enrichment analysis for the genes in the brown module. The color represents the adjusted *p*-values (BH), and the size of the spots represents the gene number.

To gain further insight into the potential functions of the 29 genes that overlapped with DEG lists and two co-expression modules, gene enrichment analysis was performed by the *clusterProfiler* package. After screening of GO enrichment analysis, we observed several enriched gene sets shown in Figure 5. The biological process (BP) of 29 genes are mainly enriched in epidermis development and epidermal cell differentiation. For the result of the cellular component (CC), it was revealed that these genes were mainly involved in apical plasma membrane, apical part of cell, and cell-cell junction. Moreover, in the molecular function (MF) analysis, peptidase regulator activity and enzyme inhibitor activity were suggested to be related to the 29 genes.

### PPI Network Construction and Hub Genes Identification

**Figure 6:**
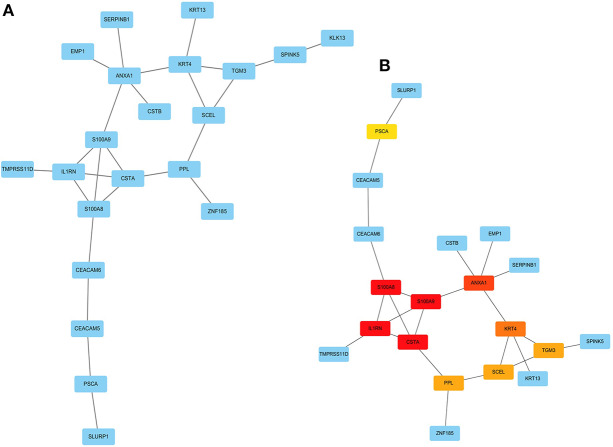
Visualization of the protein-protein interaction (PPI) network and the candidate hub genes. **(A)** PPI network of the genes between DEG lists and two co-expression modules. The blue nodes represent the genes. Edges indicate interaction associations between nodes. **(B)** Identification of the hub genes from the PPI network using maximal clique centrality (MCC) algorithm. Edges represent the protein-protein associations. The red nodes represent genes with a high MCC sores, while the yellow node represent genes with a low MCC sore.

The PPI network among the overlapped genes was established by using the STRING database, with 21 nodes and 25 edges (Figure 6A). The hub genes selected from the PPI network using the MCC algorithm of CytoHubba plugin were shown in Figure 6B. According to the MCC sores, the top ten highest-scored genes, including S100 calcium-binding protein A8 (S100A8), S100 calcium-binding protein A9 (S100A9), Interleukin-1 receptor antagonist (IL1RN), Cystatin A (CSTA), Annexin-A1 (ANXA1), Keratin 4 (KRT4), Transglutaminase 3 (TGM3), Sciellin (SCEL), Periplakin (PPL), and Prostate Stem Cell Antigen (PSCA), were selected as the hub genes.

### Verification of the Expression Patterns, the Prognostic Values, and Protein Expression of Hub Genes

**Figure 7:**
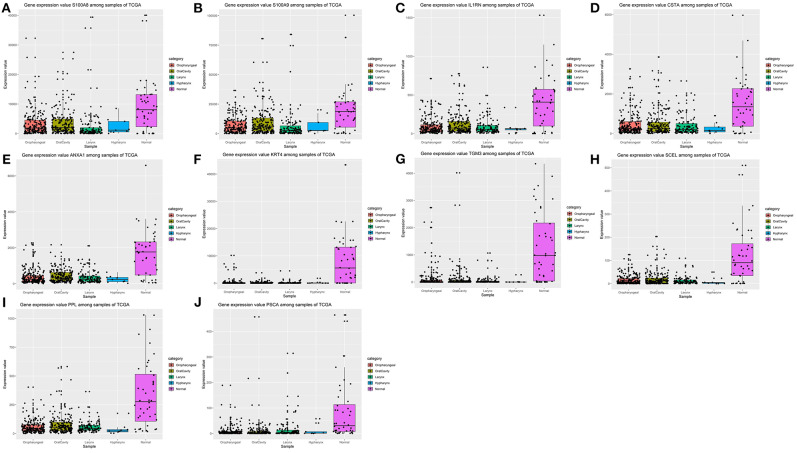
Validation of expression levels of the ten hub genes among HNSCCs and normal tissues from the TCGA database. **(A)** Gene expression value S100A8 among samples of TCGA. **(B)** Gene expression value S100A9 among samples of TCGA. **(C)** Gene expression value IL1RN among samples of TCGA. **(D)** Gene expression value CSTA among samples of TCGA. **(E)** Gene expression value ANXA1 among samples of TCGA. **(F)** Gene expression value KRT4 among samples of TCGA. **(G)** Gene expression value TGM3 among samples of TCGA. **(H)** Gene expression value SCEL among samples of TCGA. **(I)** Gene expression value PPL among samples of TCGA. **(J)** Gene expression value PSCA among samples of TCGA.

**Figure 8:**
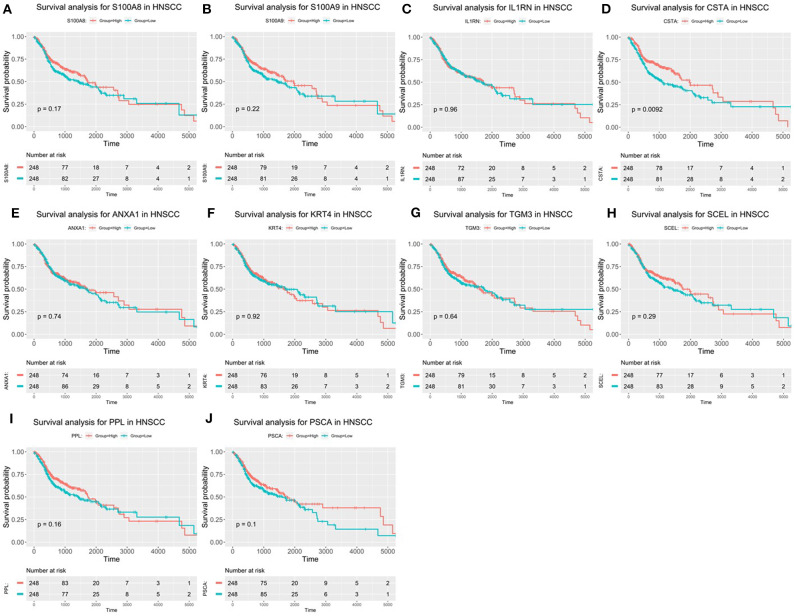
Overall survival (OS) analysis of 10 hub genes in HNSCC patients from the GEPIA2 database. **(A)** Survival analysis for S100A8 in HNSCC. **(B)** Survival analysis for S100A9 in HNSCC. **(C)** Survival analysis for IL1RN in HNSCC. **(D)** Survival analysis for CSTA in HNSCC. **(E)** Survival analysis for ANXA1 in HNSCC. **(F)** Survival analysis for KRT4 in HNSCC. **(G)** Survival analysis for TGM3 in HNSCC. **(H)** Survival analysis for SCEL in HNSCC. **(I)** Survival analysis for PPL in HNSCC. **(J)** Survival analysis for PSCA in HNSCC. The patients were stratified into high-level group (red) and low-level group (green) according to median expression of the gene. Log-rank *P* < 0.05 was considered to be a statistically significant difference.

**Figure 9:**
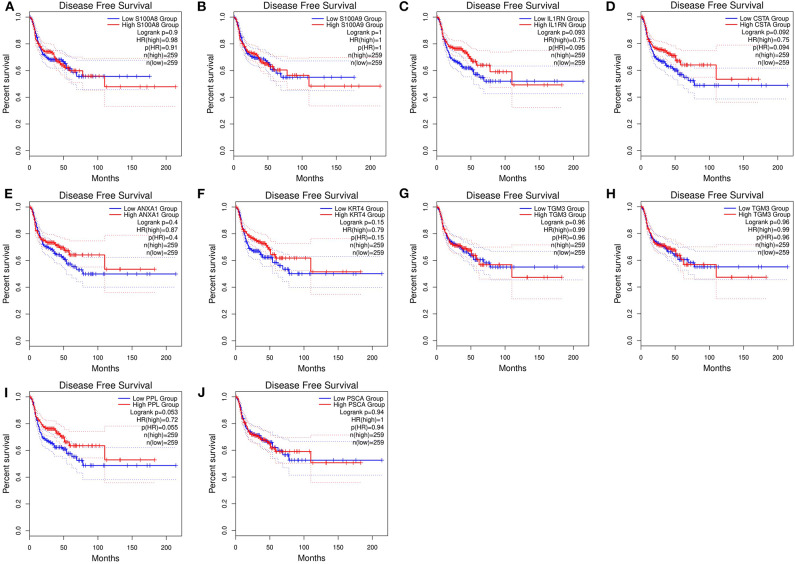
Disease-free survival (DFS) analysis of 10 hub genes in HNSCC patients from the GEPIA2 database. **(A)** Survival analysis for S100A8 in HNSCC. **(B)** Survival analysis for S100A9 in HNSCC. **(C)** Survival analysis for IL1RN in HNSCC. **(D)** Survival analysis for CSTA in HNSCC. **(E)** Survival analysis for ANXA1 in HNSCC. **(F)** Survival analysis for KRT4 in HNSCC. **(G)** Survival analysis for TGM3 in HNSCC. **(H)** Survival analysis for SCEL in HNSCC. **(I)** Survival analysis for PPL in HNSCC. **(J)** Survival analysis for PSCA in HNSCC. The patients were stratified into high-level group (red) and low-level group (green) according to median expression of the gene. Log-rank *P* < 0.05 was considered to be a statistically significant difference.

**Figure 10:**
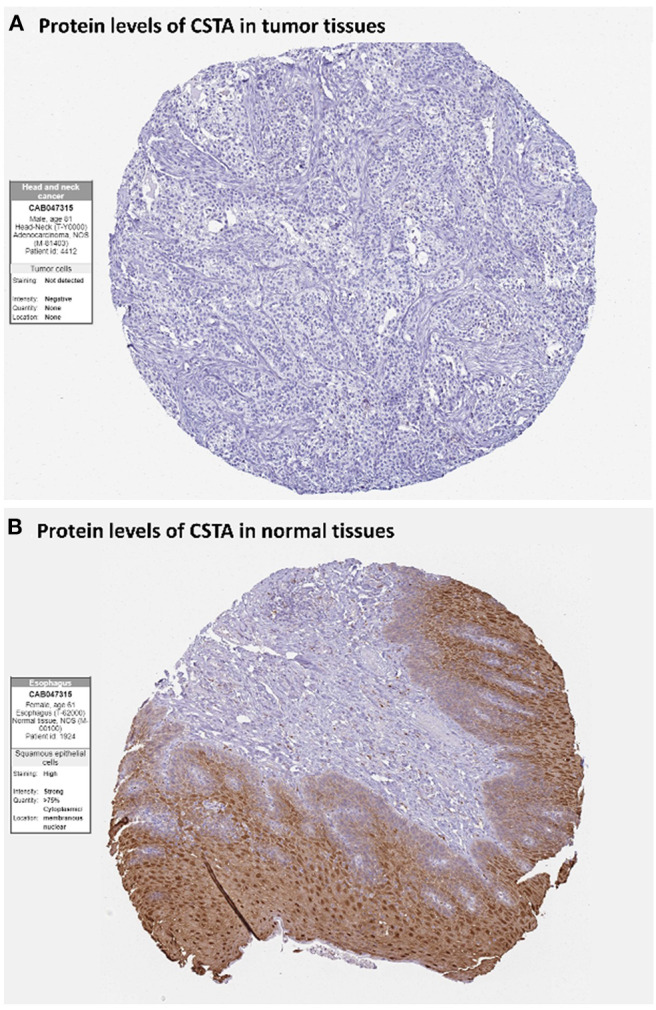
Immunohistochemistry of the CSTA gene in HNSCC and normal tissues from the human protein atlas (HPA) database. **(A)** Protein levels of CSTA in HNSCC tissues (antibody CAB047315; staining: not detected; intensity: negative; quantity: none). **(B)** Protein levels of CSTA in normal oral mucosa tissues (antibody CAB047315; staining: high; intensity: strong; quantity: >75%).

After the ten hub genes (S100A8, S100A9, IL1RN, CSTA, ANXA1, KRT4, TGM3, SCEL, PPL, and PSCA) were screened out by CytoHubba plugin, we verified the expression levels of the hub genes among the patients of the TCGA database. As shown in Figure 7, all of the ten hub genes were found to be significantly downregulated in HNSCC carcinoma compared with normal tissues. In addition, OS and DFS analyses of the ten hub genes were performed by Kaplan–Meier plotter using the R *survival* package (Figure 8) and the GEPIA2 database (Figure 9) for investigating the prognostic values of the hub gens in the HNSCC patients. Of the ten hub genes, the Kaplan–Meier analyses suggested that the lower expression level of CSTA was significantly associated with worse OS of the HNSCC patients (*P* < 0.05) (Figure 8D), while with DFS there was no significant difference observed in HNSCC patients with an expression level of CSTA (*P* < 0.05) (Figure 9D). Furthermore, the protein levels of the CSTA gene was significantly lower in tumor tissues compared with normal tissues based on the HPA database (Figure 10). All the above-mentioned observations confirmed down-expression of CSTA is associated with worse prognosis and lower overall survival in HNSCC patients.

## Discussion

Head and neck squamous cell carcinomas (HNSCC) are a group of cancers found in several regions, including the mouth, nose, throat, larynx, sinuses, or salivary glands. Although the treatment of head and neck cancer has improved, the prognosis of patients is generally poor due to the lack of precise molecular targets. Therefore, better biomarkers for specific prognosis and progression of HNSCC are demanded. In this study, a total of 29 significant genes with the same expression trends were identified in the TCGA and GSE6631 databases using integrated bioinformatic analysis. As suggested in functional annotation analysis by the *clusterProfiler* package, these genes were mainly enriched in epidermis development and differentiation, which are basic processes in cell proliferation. Furthermore, according to MCC scores from the CytoHubba plugin in Cytoscape, the top 10 HNSCC-related genes were screened out (namely S100A8, S100A9, IL1RN, CSTA, ANXA1, KRT4, TGM3, SCEL, PPL, and PSCA) and all their expression patterns were found be downregulated in HNSCC tissues compared with the normal controls. Among them, CSTA downexpression was significantly associated with poor overall survival in head and neck cancers. Finally, survival and immunohistochemical analysis for CSTA was carried out.

CSTA, also known as Cystatin A or stefin A, is a member of the cystatin superfamily. It is an intracellular inhibitor regulating the activities of cystatin proteinase and has an important role in desmosome-mediated cell-cell adhesion (29, 30). Furthermore, lower mRNA levels of CSTA have been reported in breast (31), prostate (32), skin (30), and esophagus tumors (33) as compared to adjacent control tissues (34, 35). In our study, CSTA was down-regulated in tumor tissues compared with normal tissues, showing a significant correlation with HNSCC. Previous studies demonstrated that higher levels of CSTA in tumor tissues have been shown to correlate with a favorable prognosis of patients with HNSCC, that was consistent with our finding of survival analysis (36–39).

As with all research, our study also had limitations about the classification of tumors to different subtypes. Although we provided a comprehensive bioinformatics analysis to identify potential diagnostic genes between cancer and normal tissues, it may not be very accurate for each patient with HNSCC subtypes. Moreover, the molecular mechanisms involved in the survival-related genes that affected the prognosis of HNSCC patients should be further validated through a series of experiments.

In summary, by integrating WGCNA with differential gene expression analysis, our study generated the significant survival-related gene CSTA that has potential for prognosis prediction in HNSCC.

## Data Availability Statement

All available data were analyzed in this study. Those can be found here: TCGA (https://portal.gdc.cancer.gov/), GEO (https://www.ncbi.nlm.nih.gov/gds), and HPA (https://www.proteinatlas.org/).

## Author Contributions

J-HC, CW, and CL: conceptualization and methodology. CW and J-HC: software and data curation. JT and CL: validation. J-HC and CL: writing—original draft preparation. CW and JT: writing—review, editing, and supervision.

## Conflict of Interest

The authors declare that the research was conducted in the absence of any commercial or financial relationships that could be construed as a potential conflict of interest.
